# Effects of the walk ratio on the effectiveness of mechanical energy utilization during gait in healthy young people

**DOI:** 10.1098/rsos.250740

**Published:** 2025-09-03

**Authors:** Toru Sakuma, Kensaku Kimura, Mika Konishi

**Affiliations:** ^1^ Tsukuba University of Technology, Tsukuba, Japan

**Keywords:** gait, walk ratio, cadence, step length, joint torque, power

## Abstract

When walking freely, humans prefer maintaining a nearly constant walk ratio (WR) (step length/cadence). An unnatural WR requires greater metabolic energy expenditure, with mechanical power demands underlying the metabolic response. To investigate the effects of WR on the effectiveness of mechanical energy utilization during walking, this study compared the average total absolute mechanical power during the stance (${\overset{-}{P}}_{\text{total, stance}}$) and swing (${\overset{-}{P}}_{\text{total, swing}}$) phases between preferred and unnatural WRs at a fixed walking speed. Twenty healthy participants walked at their preferred WR and at six types of unnatural WRs at a preferred walking speed. The unnatural WRs comprised six cadence conditions (±10, ±15 and ±20 of preferred cadence). ${\overset{-}{P}}_{\text{total, stance}}$ was significantly higher at high WRs (slower cadence) than at the preferred WR (*p* < 0.05) owing to an increased braking ground reaction force and shorter relative stance phase duration. ${\overset{-}{P}}_{\text{total, swing}}$ was significantly higher at low WRs (faster cadence) than at the preferred WR (*p* < 0.05) owing to the hip and knee joint powers being applied strongly and synchronously to prevent excessive knee flexion and extension. The preferred WR can optimize the effectiveness of mechanical work production, which may help to minimize metabolic energy expenditure.

## 1. Introduction

Humans can freely choose from a wide range of cadences (i.e. number of steps per minute) and step length combinations for a given walking speed. Nevertheless, when walking freely, healthy people prefer to keep their walk ratio (WR) (step length/cadence) at around 0.006 to 0.007 (m step^−1^ min^−1^) [1]. Walking economy is optimized at the preferred WR, and when an unnatural WR is enforced as an experimental protocol, the metabolic energy cost increases in healthy people [2–6], obese younger people [7] and obese elderly people [8].

The effects of changes in the step length and cadence on mechanical power have been studied in the past. Longer step length and step width required more mechanical work to change the centre of mass (CoM) velocity downwards to upwards during the transition from step to step [9–11]. The mechanical internal power to accelerate the limbs relative to the CoM increases with increase in step frequency [12]. The net metabolic rate for leg swinging has been approximated with the fourth power of frequency and in relation to a hypothesized cost of force production for short durations [13].

It has also been shown that deviating from a preferred WR has a negative effect on mechanical efficiency (i.e. the ratio of mechanical work, e.g. joint work, to metabolic cost). Umberger & Martin [6] reported that metabolic cost was minimized at the preferred stride rate but that mechanical power and efficiency showed plateaus on opposite sides of the preferred stride rate. Deviation from a preferred WR reduces the amount of elastic energy that can be stored and released in the ankle plantar flexors; walking at a slower cadence increased the shortening of medial gastrocnemius muscle fascicles during positive ankle power production and walking at a faster cadence had greater cumulative activation of plantar flexors [14]. The mechanical work performed by the muscle to compensate for the less effective elastic mechanism requires more metabolic energy, resulting in a decreased mechanical efficiency during walking.

Although mechanical efficiency is essential for economical walking, mechanical efficiency alone cannot sufficiently explain walking economy. An increase in the metabolic cost of locomotion depends on the total amount of mechanical work required to move the body forward a given distance [15–18]. In other words, even if muscles operate at optimal muscle efficiency, if they need do more work to travel a given distance the energy cost will increase. Indeed, although there was no difference in mechanical efficiency between normal walking and bouncy walking (increasing the vertical CoM displacement), the latter increased the metabolic energy cost [19]. When comparing different gaits at a fixed walking speed, a lower total mechanical power indicates effective propulsion of the body and vice versa. In relation to unnatural WR, how effectively mechanical work is used during the stance phase to propel the CoM forward and how effectively mechanical work is used during the swing phase to carry the foot forward will provide new insight into the reason why metabolic energy expenditure increases with unnatural WR at a fixed walking speed.

The purpose of the present study was to investigate the effects of different WRs at a fixed walking speed on the effectiveness of mechanical work production in healthy individuals. In a previous study, time-series data of joint power (JP) at different WR showed that a high WR (i.e. slower cadence and longer step length combination) required more negative power at the knee and positive power at the ankle during the stance phase, and that a low WR (i.e. faster cadence and shorter step length combination) required more positive power at the hip and negative power at the knee during the swing phase [6]. In line with this previous research, our study tested two hypotheses: hypothesis 1 was that the effectiveness of mechanical work production during stance would be impaired for a high WR compared with the preferred WR; hypothesis 2 was that the effectiveness of mechanical work of limb swing would be impaired for a low WR compared with the preferred WR.

## 2. Material and methods

### 2.1. Sample size calculation

An *a priori* power analysis was calculated using GPower3 [20]. According to Cohen’s *f* value as a measure of effect size, 0.25 represent a medium effect [21]. Seventeen participants were required to detect an effect size *f* of 0.25 with a power of 0.8, an alpha of 0.05, a number of groups of 1, a number of measurements of 7, a correlation among repeated measures of 0.5 and a nonsphericity correction *ε* of 1.

### 2.2. Participants

Twenty healthy people participated in the present study (13 men and seven women; mean age ± s.d., 24.4 ± 3.3 years; height, 1.71 ± 0.08 m; mass, 65.7 ± 12.9 kg). None of the participants had a neuromusculoskeletal disease that would affect gait or cognition. The study was approved by the Ethics Committee of Tsukuba University of Technology (no. 202304), and all participants provided written informed consent to participate.

### 2.3. Data collection

Initially, to determine each participant’s preferred WR (i.e. the preferred step length and cadence combination) at an individual’s self-selected preferred walking speed, all participants were asked to walk naturally on a 15 m walkway. By ‘naturally’, we mean that the participant was walking freely without any external instruction. We recorded the time and number of steps taken to walk 10 m, excluding the first 2.5 m and the last 2.5 m of the 15 m walkway, to calculate step length and cadence during a steady-state walk. Cadence was a step frequency calculated as the number of steps per time for the 10 m walk, and step length was a distance calculated as 10 m divided by the number of steps. This was repeated five times, and the average of the five values was used as the individual’s preferred cadence and step length in an experimental protocol. The coefficient of variation for the individual’s self-selected preferred walking speed over the five different trials was 2.8% on average for all participants.

The preferred WR was defined as the ratio of the preferred step length to cadence, and an unnatural WR was defined as the ratio of a longer step length to a slower cadence or the ratio of a shorter step length to a faster cadence. Because the denominator of WR is cadence, a combination of faster cadence and shorter step length represents a low WR and a combination of slower cadence and longer step length represents a high WR. To compare preferred WR with a different unnatural WR, under the condition that the walking speed always remained at the individual’s self-selected preferred speed, even when the WR was changed, the experimental protocol included preferred WR, three types of low WRs and three types of high WRs. These WRs were defined using seven different cadences, which were decided on the basis of the preferred cadence (preferred): preferred, ±10% of preferred, ±15% of preferred and ±20% of preferred. To establish a constant speed, step length was also controlled in relation to the seven different cadence conditions. For the experimental protocol, all participants were asked to walk the 15 m overground walkway while matching their cadence to the pitch of a metronome set at the desired frequency and matching their step length to the line markings for the walkway floors [6,22,23]. Before collecting any data, participants practised each experimental condition several times until they could walk while looking down at the floor as little as possible. To confirm that participants were able to walk at the cadence and step length defined according to the experimental conditions, all trials were filmed from the side of the walkway using an iPad (Apple Inc., Cupertino, CA, USA). If the participant’s heel contact position was more than 5 cm away from the floor mark or if the timing of the heel contact did not match the metronome sound, the trial was considered a failure (this decision was made jointly by two researchers based on the video and audio material on the iPad). All the participants performed the cadence conditions −20, −15, −10%, preferred, +10, +15 and +20% in sequence. Each cadence condition was repeated until five successful trials were obtained, and the average of these five values was used in subsequent statistical analysis. In total, approximately 80 trials were performed to obtain the 35 (seven conditions × five trials) successful trials for each participant, and the walking portion of the protocol took approximately 70 min (table 1).

**Table 1.** Spatiotemporal parameters for the seven different cadence conditions. (Values are means ± s.d. Step length has been normalized to body height.)

cadence conditions	−20%	−15%	−10%	preferred	+10%	+15%	+20%
speed (m s^−1^)	1.49 ± 0.19	1.48 ± 0.18	1.47 ± 0.19	1.47 ± 0.19	1.46 ± 0.18	1.46 ± 0.19	1.45 ± 0.19
cadence (steps min^−1^)	93.4 ± 6.3	98.5 ± 6.1	104.1 ± 6.5	115.5 ± 7.3	125.9 ± 7.8	131.9 ± 8.9	137.4 ± 9.2
step length (m height^−1^)	0.56 ± 0.04	0.52 ± 0.04	0.49 ± 0.04	0.45 ± 0.04	0.41 ± 0.03	0.39 ± 0.03	0.37 ± 0.03
walk ratio (m min steps^−1^)	0.0102 ± 0.0009	0.0091 ± 0.0008	0.0081 ± 0.0007	0.0066 ± 0.0006	0.0055 ± 0.0005	0.0050 ± 0.0004	0.0046 ± 0.0004

Reflective spherical markers were placed on 47 anatomical landmarks of the body. To define a link-segment model, 15 segments (head, upper torso, lower torso and extremities (arms, forearms, hands, thighs, shanks and feet)) were defined based on these landmarks. Joint centres for the extremities were identified using the landmarks for both sides of each joint, and the centre of the hip joint was estimated based on the locations of the anterior superior iliac spine and the greater trochanter [24]. All participants wore the same type of running shoes (GEL-MOOGEE, ASICS Inc., Tokyo, Japan) during the test, so that foot markers were placed on the surface of the shoes. Marker trajectories were recorded using a VICON motion analysis system (Vicon Motion Systems Ltd., Oxford, UK) with nine Bonita cameras sampling at a rate of 200 Hz. Ground reaction forces were measured using a force platform (type 9260AA6; Kistler Instrument Corp., Winterthur, Switzerland) placed in the centre of the walkway, operating at 1000 Hz. All participants stepped on the force platform with their left-side foot.

### 2.4. Data analysis

#### 2.4.1. Link-segment model of the whole body

All data were analysed using MATLAB (R2019b; MathWorks, Natick, MA, USA). The coordinate data were smoothed using a Butterworth digital filter with optimal cut-off frequencies, determined using the residual error method proposed by Winter [25] and ranged from 7.25 to 15 Hz. The local coordinate systems for the 15 segments were defined as a right-handed orthogonal reference frame based on the distal joint centre for each segment, whereas those for the head, hand and foot were defined based on the proximal joint centre. The values for segment mass, CoM and moment of inertia were derived based on data reported for young Japanese athletes [26]. The CoM of the whole body was calculated as a weighted average accounting for the masses and positions of the 15 segments.

#### 2.4.2. Spatiotemporal parameter

The range for analysis was the period from an initial contact to the next initial contact on the left foot (i.e. one gait cycle on the left side). The moment of foot contact with the ground was defined as the first instance of the vertical component of the ground reactions force (GRF). Stride length was the distance the left heel moved forward in one gait cycle and step length was half the stride length. Cadence was the reciprocal of half of one gait cycle time. Walking speed was obtained by dividing stride length by one gait cycle time. WR was the ratio of step length to cadence [1]. Duty factor was the ratio of stance phase time to one gait cycle time [27,28].

#### 2.4.3. Joint kinematics and kinetics

Hip, knee and ankle joint angles were calculated using a joint coordinate system [29]. Joint angular velocity (JAV) was calculated as the time derivative of the joint angle. Three-dimensional joint torques (JTs) at the ankle, knee and hip joints were calculated through inverse dynamics [25], and JP was calculated as the inner product of the JAV and JT. Mechanical work performed by the torques at the hip, knee and ankle joints was calculated as the time integration of the instantaneous positive and negative JPs. The mechanical work was calculated for the stance phase and swing phase, respectively. $W_{j,\;\text{stance}}$, ${PW}_{j,\;\text{stance}}$ and ${NW}_{j,\;\text{stance}}$ were the absolute, positive and negative work done by joint *_j_* during the stance phase, respectively. $W_{j,\;\text{swing}}$, ${PW}_{j,\;\text{swing}}$ and ${NW}_{j,\;\text{swing}}$ were the absolute, positive and negative work done by joint *_j_* during the swing phase, respectively.

To assess the effectiveness of the mechanical work production, we calculated the amount of mechanical work performed per unit time. This metric is defined as the average mechanical power. Because speed is kept constant in our experiment, the average mechanical power will capture both the effect of differences in the amount of work per step (including limb swing) as well as differences in the horizontal distance travelled per step. The average absolute power (${\overset{-}{P}}_{j}$), average positive power (${\overset{-}{P}}_{j}^{+}$) and average negative power (${\overset{-}{P}}_{j}^{-}$) were computed by dividing each work expression by the stance or swing phase time. ${\overset{-}{P}}_{\text{total, stance}}$ and ${\overset{-}{P}}_{\text{total, swing}}$ were the sum of the average absolute power at the left hip joint in the sagittal, frontal and transverse planes, at the left knee joint in the sagittal plane and at the left ankle joint in the sagittal plane during the stance phase and swing phase, respectively. A boundary between the stance and swing phases was defined as the moment when the vertical GRF was less than one-third of the body weight. Note that this did not correspond to the full stance phase time. The basis for the definition of the boundary is based on Perry & Burnfield [30], who refer to the last phase of the stance phase as the pre-swing. This is because, during this phase, the functional role of the lower limb joints shifts from weight bearing to preparation for the forward swing. To clarify the contribution of mechanical work to the CoM movement and lower limb swing, our study adopted this boundary to calculate mechanical work separately between the stance and swing phases.

### 2.5. Statistical analysis

The Shapiro–Wilk test was performed to determine the normality of the data. The overall effects of cadence condition on ${\overset{-}{P}}_{\text{total, stance}}$ and ${\overset{-}{P}}_{\text{total, swing}}$ were tested using one-way repeated-measures analysis of variance (ANOVA) for normally distributed variables and the Friedman test for non-normally distributed variables. Dunnett’s test was used for multiple comparisons of the preferred condition with the ±10, ±15 and ±20% conditions. These statistical analyses were conducted using SPSS Statistics (v. 29.0.2; IBM Inc., Armonk, NY, USA). Repeated measures correlation (rmcorr) coefficients were calculated to test the strength of the relationship between WR and duty factor using the rmcorr package [31] in RStudio (v. 4.4.2; Boston, MA, USA). The level of statistical significance was considered to be *p* < 0.05.

## 3. Results

**Figure 1.**
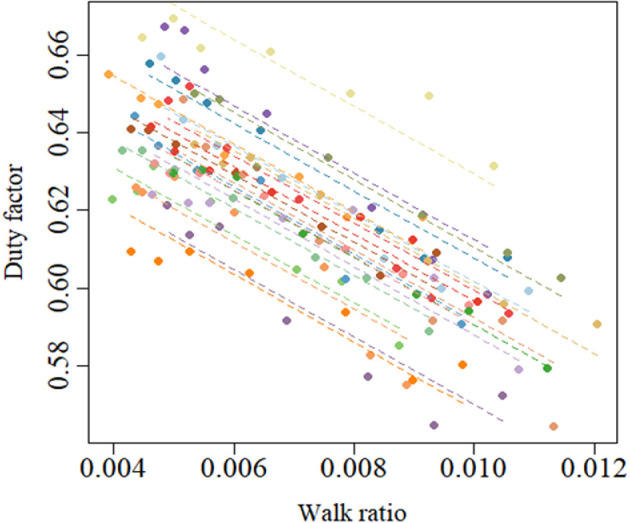
Relationship between the walk ratio and duty factor.

**Figure 2.**
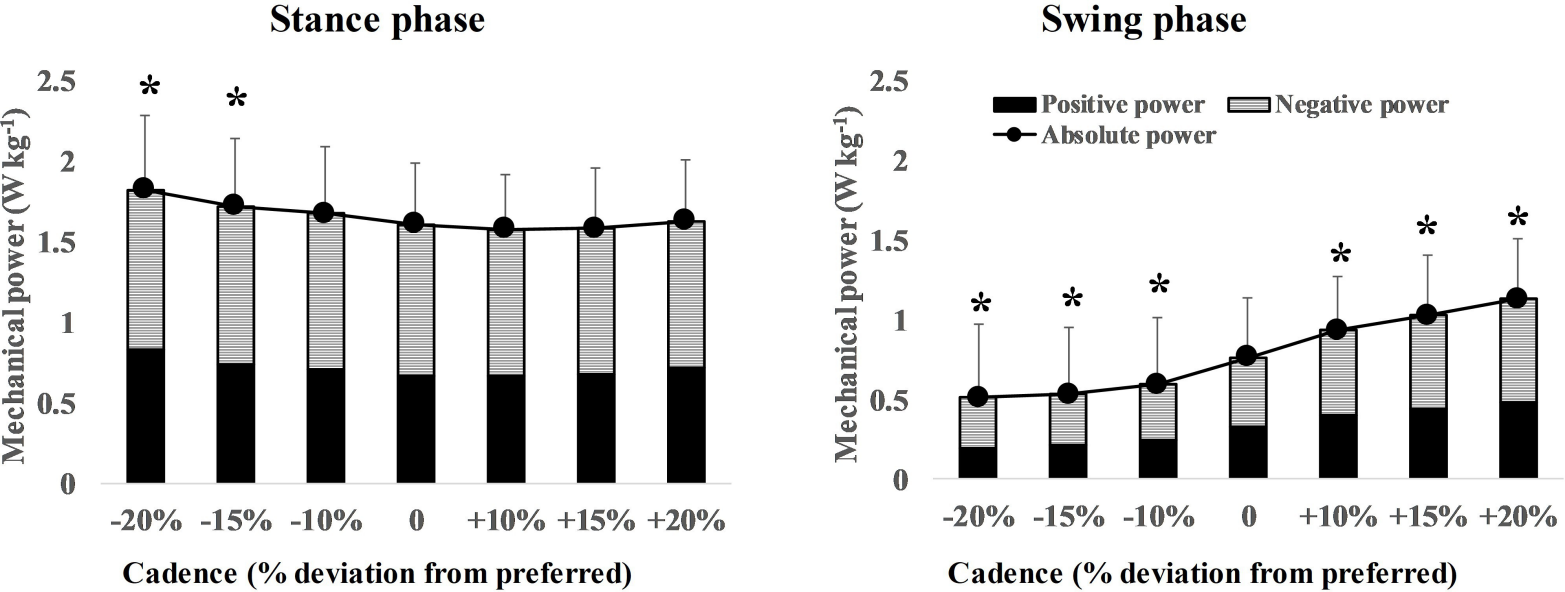
Changes in the average total absolute mechanical power during the stance (${\overset{-}{P}}_{\text{total, stance}}$) and swing (${\overset{-}{P}}_{\text{total, swing}}$) phases according to the unnatural WRs. High and low WR were represented by slow and fast cadence conditions, respectively. During the stance phase, ${\overset{-}{P}}_{\text{total, stance}}$ increased for high WR (slow cadence). During the swing phase, ${\overset{-}{P}}_{\text{total, swing}}$ gradually increased from high WR (slow cadence) to low WR (fast cadence). Average total absolute mechanical power was the sum of positive and negative power over the hip, knee and ankle joints. Values are means ± s.d. Mechanical power has been normalized to body mass. Asterisks indicate a significant difference (*p* < 0.05) from the preferred value as determined by a Dunnett’s post hoc test.

**Figure 3.**
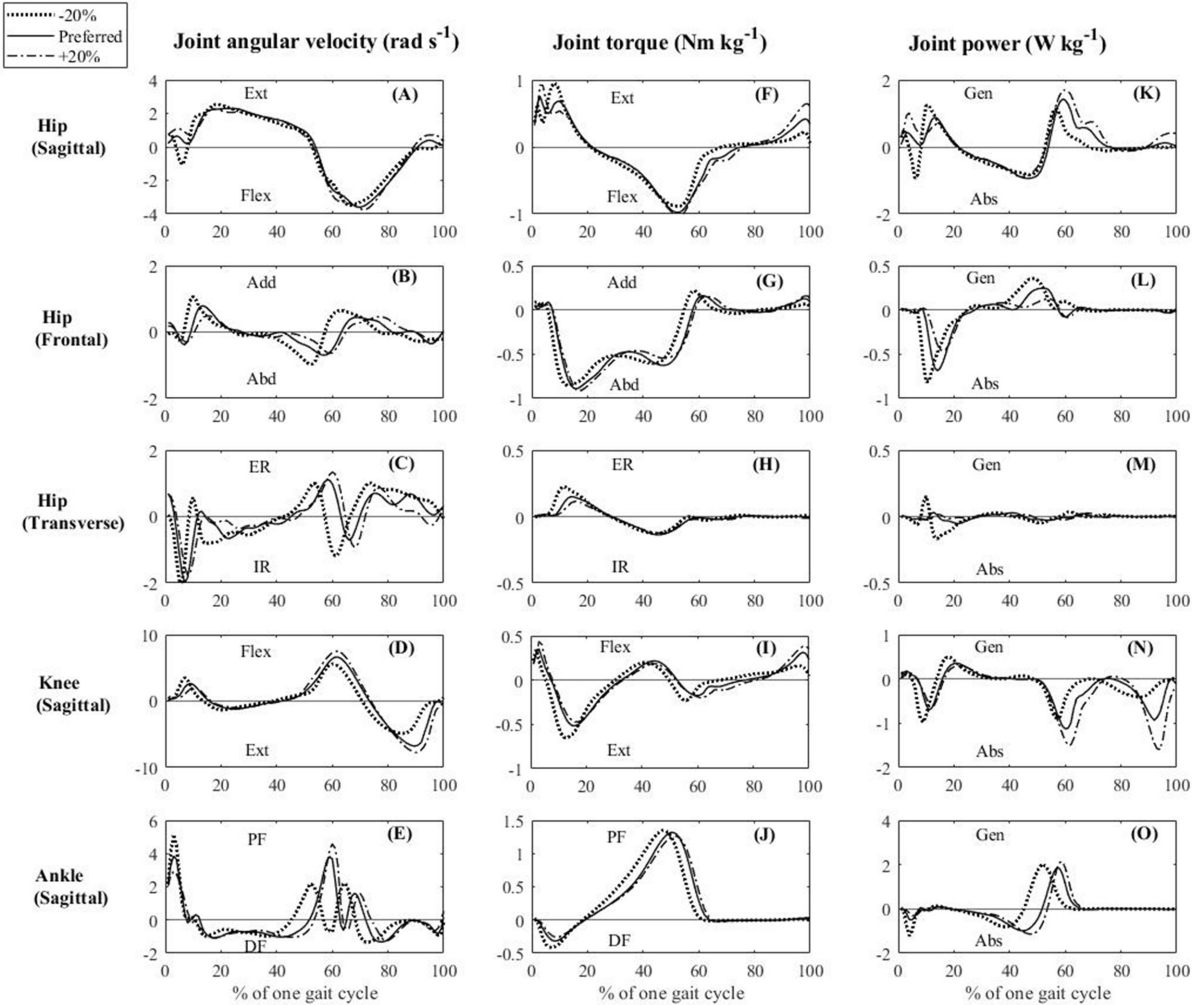
Averaged time series data of joint angular velocity (A–E), joint torque (F–J) and joint power (K–O) during one gait cycle in the −20%, preferred and +20% cadence conditions. The ±10% and ±15% cadence conditions have been omitted for clarity but were generally intermediate to the preferred and ±20% cadence conditions. Joint torque and joint power have been normalized to body mass. At the −20% cadence condition, greater torque and negative power were produced at the hip, knee and ankle joints during the early stance phase (0% to approx. 20% of one gait cycle), and greater torque and positive power were produced at the ankle joint during the late stance phase (approx. 40–60% of one gait cycle). At the +20% cadence condition, greater positive power was exerted by the hip flexion torque and greater negative power was exerted by the knee extension torque during the pre- to early swing phase (approx. 60–80% of one gait cycle), while greater positive power was exerted by the hip extension torque and greater negative power was exerted by the knee flexion torque during the late swing phase (approx. 80–100% of one gait cycle). Ext, extension; Flex, flexion; Add, adductor; Abd, abductor; ER, external rotation; IR, internal rotation; PF, plantarflexion; DF, dorsiflexion; Gen, generation; Abs, absorption.

The duty factor had a significant negative correlation (*r_rm_* = −0.95, 95% confidence interval: −0.96 to −0.93, *p* < 0.001) with the WR (figure 1). The cadence condition had significant effects on ${\overset{-}{P}}_{\text{total, stance}}$ (*F*_2, 36_ = 19.40, *p* < 0.001) and ${\overset{-}{P}}_{\text{total, swing}}$ (*$\chi_{6}^{2}$* = 112.8, *p* < 0.001). Multiple comparisons of the preferred condition with the −20 and −15% conditions (i.e. high WR) showed that ${\overset{-}{P}}_{\text{total, stance}}$ was significantly greater (*p* < 0.05). In comparison with the +20, +15 and +10% conditions (i.e. low WR), ${\overset{-}{P}}_{\text{total, swing}}$ was significantly greater (*p* < 0.05; figure 2). The patterns of the change in JAV (figure 3A–E), JT (figure 3F–J) and JP (figure 3K–O) during one gait cycle varied with the cadence condition. The moments when the vertical GRF was less than one-third of the body weight were, as the per cent of one gait cycle, 56% for the −20% cadence, 58% for the preferred cadence condition and 59% for the +20% cadence, respectively. The joint with the greatest positive power during the stance phase was the ankle joint, followed by the hip (sagittal) joint, and the negative power for the hip (sagittal) and ankle joints was greater than that for the other joints. During the swing phase, only the positive power for the hip (sagittal) joint and the negative power for the knee joint were noticeable, while the power for the other joints was very low under all experimental conditions (table 2).

**Table 2.** Average mechanical power during the stance and swing phases for the seven different cadence conditions. (sagitt, sagittal plane; front, frontal plane; trans, transverse plane; pos, average positive power; neg, average negative power. Values are means ± s.d. Power values have been normalized to body mass.)

power (W kg^−1^)		cadence conditions						
		−20%	−15%	−10%	preferred	+10%	+15%	+20%
stance phase								
hip (sagitt)	pos	0.23 ± 0.13	0.22 ± 0.10	0.22 ± 0.11	0.24 ± 0.11	0.27 ± 0.10	0.29 ± 0.12	0.32 ± 0.13
	neg	−0.37 ± 0.10	−0.35 ± 0.10	−0.34 ± 0.10	−0.32 ± 0.10	−0.29 ± 0.09	−0.28 ± 0.09	−0.28 ± 0.09
hip (front)	pos	0.10 ± 0.05	0.09 ± 0.05	0.09 ± 0.05	0.07 ± 0.05	0.07 ± 0.04	0.06 ± 0.04	0.06 ± 0.04
	neg	−0.13 ± 0.07	−0.14 ± 0.07	−0.13 ± 0.08	−0.12 ± 0.07	−0.10 ± 0.07	−0.09 ± 0.07	−0.09 ± 0.07
hip (trans)	pos	0.02 ± 0.01	0.02 ± 0.01	0.02 ± 0.01	0.01 ± 0.01	0.01 ± 0.01	0.01 ± 0.01	0.01 ± 0.01
	neg	−0.04 ± 0.02	−0.04 ± 0.02	−0.03 ± 0.02	−0.02 ± 0.01	−0.02 ± 0.01	−0.02 ± 0.01	−0.02 ± 0.01
knee (sagitt)	pos	0.13 ± 0.06	0.12 ± 0.07	0.12 ± 0.07	0.12 ± 0.06	0.12 ± 0.05	0.11 ± 0.05	0.11 ± 0.04
	neg	−0.16 ± 0.10	−0.16 ± 0.10	−0.16 ± 0.10	−0.17 ± 0.09	−0.19 ± 0.10	−0.20 ± 0.13	−0.21 ± 0.13
ankle (sagitt)	pos	0.34 ± 0.12	0.29 ± 0.12	0.26 ± 0.11	0.21 ± 0.10	0.20 ± 0.08	0.20 ± 0.08	0.21 ± 0.09
	neg	−0.29 ± 0.04	−0.29 ± 0.04	−0.29 ± 0.04	−0.31 ± 0.04	−0.31 ± 0.06	−0.32 ± 0.06	−0.32 ± 0.06

swing phase								
hip (sagitt)	pos	0.13 ± 0.07	0.14 ± 0.06	0.17 ± 0.07	0.24 ± 0.08	0.30 ± 0.10	0.34 ± 0.11	0.38 ± 0.14
	neg	−0.05 ± 0.03	−0.04 ± 0.03	−0.04 ± 0.03	−0.03 ± 0.02	−0.03 ± 0.02	−0.03 ± 0.02	−0.04 ± 0.03
hip (front)	pos	0.02 ± 0.01	0.02 ± 0.01	0.01 ± 0.01	0.01 ± 0.01	0.01 ± 0.01	0.01 ± 0.01	0.01 ± 0.01
	neg	−0.01 ± 0.01	−0.01 ± 0.01	−0.01 ± 0.01	−0.02 ± 0.01	−0.02 ± 0.01	−0.02 ± 0.01	−0.02 ± 0.01
hip (trans)	pos	0.01 ± 0.00	0.01 ± 0.00	0.01 ± 0.00	0.01 ± 0.00	0.01 ± 0.00	0.01 ± 0.00	0.01 ± 0.00
	neg	−0.00 ± 0.00	−0.00 ± 0.00	−0.00 ± 0.00	−0.00 ± 0.00	−0.00 ± 0.00	−0.00 ± 0.00	−0.01 ± 0.00
knee (sagitt)	pos	0.02 ± 0.01	0.02 ± 0.01	0.02 ± 0.02	0.03 ± 0.03	0.03 ± 0.03	0.03 ± 0.04	0.03 ± 0.04
	neg	−0.23 ± 0.06	−0.25 ± 0.06	−0.29 ± 0.07	−0.37 ± 0.09	−0.46 ± 0.12	−0.52 ± 0.14	−0.58 ± 0.17
ankle (sagitt)	pos	0.02 ± 0.02	0.03 ± 0.02	0.04 ± 0.02	0.05 ± 0.02	0.05 ± 0.01	0.05 ± 0.02	0.06 ± 0.02
	neg	−0.02 ± 0.01	−0.01 ± 0.01	−0.01 ± 0.01	−0.01 ± 0.00	−0.01 ± 0.00	−0.01 ± 0.00	−0.01 ± 0.01

## 4. Discussion

The present study found that the ${\overset{-}{P}}_{\text{total, stance}}$ increased with an increased WR and that the ${\overset{-}{P}}_{\text{total, swing}}$ increased with a decreased WR (figure 2); therefore, the first and second hypotheses were supported. Our findings on the relationship between WR and the effective use of joint work offer a reasonable explanation for the previously reported dependence of metabolic energy expenditure on the WR [3,5–8]. Several previous studies have shown that metabolic energy responded in a U-shaped manner to changes in cadence at a fixed speed. The bottom of the U-shape appeared around the preferred cadence, but the U-shape was not perfectly symmetrical: the slope towards smaller cadences (higher WRs) was steeper than the slope towards greater cadences (low WRs) [3,5,6]. In the present study, the increase in the ${\overset{-}{P}}_{\text{total, stance}}$ at high WRs may be one reason for the steep increase in metabolic power at high WRs. It may seem that there is a trade-off between the increase in the ${\overset{-}{P}}_{\text{total, stance}}$ and the decrease in the ${\overset{-}{P}}_{\text{total, swing}}$ at high WRs, but this trade-off is unlikely to improve the walking economy at high WRs. This is because the magnitude of mechanical power was greater during the stance phase than during the swing phase (figure 2). Since metabolic energy expenditure increases in a curvilinear fashion with increasing mechanical power [32,33], it is suggested that walking at high WR would require more metabolic energy owing to the increased ${\overset{-}{P}}_{\text{total, stance}}$, resulting in lower walking economy. On the other hand, the increase in the ${\overset{-}{P}}_{\text{total, swing}}$ at low WRs may be a reason for the slight increase in metabolic power at low WRs. This is because the ${\overset{-}{P}}_{\text{total, stance}}$ did not differ between the low and preferred WRs. Our findings are consistent with a recent musculoskeletal modelling study which reported that self-selected stride frequency corresponds with reducing the mechanical demands placed on muscles [34], supporting previous studies showing that preferred WR corresponds to a reduction in metabolic energy expenditure [3,5–8].

This study examined the average mechanical power separately in the stance and swing phases, not over one gait cycle. A feature of this approach is that it can quantify how effectively the JP in the lower limb joints has been used for CoM propulsion or lower limb swing, respectively. Umberger & Martin [6] calculated the mechanical power based on the inverse dynamics method in a similar way to our study but calculated the average mechanical power over one gait cycle. Their results showed that the average mechanical power for the high WR was smaller than that for the preferred WR, which is the opposite of our results. The discrepancy depends on the period used to calculate the average mechanical power. In the present study, the ${\overset{-}{P}}_{\text{total, stance}}$ was calculated by dividing the mechanical work produced during the stance phase by its duration, and the ${\overset{-}{P}}_{\text{total, swing}}$ was calculated by dividing the mechanical work produced during the swing phase by its duration. Observing the pattern of change in the lower limb JPs during gait (figure 3K–O), lower limb joints, except the knee, showed greater JP during the stance phase than during the swing phase, which is consistent with previous studies [6,35–37]. If the average power is calculated over one gait cycle, it should be lower than the actual average power in the stance phase. In addition, the difference between the average mechanical power during one gait cycle and during the stance phase should increase when the duty factor is small. Our results showed a significant negative correlation between the duty factor and WR (figure 1). Therefore, it is appropriate to calculate the average mechanical power for the stance and swing phases separately, rather than for one gait cycle, when comparing the average mechanical power between different WRs.

Analysis of each joint separately revealed a trend for the average positive and negative power in the stance phase to increase as cadence decreased (table 2). In terms of patterns of changes in JPs (figure 3K–O), in the −20% cadence condition, the negative powers of the hip extension, abduction, external rotation, knee extension and ankle dorsiflexion torque during the early stance phase were greater while, during the late stance phase, the positive power of ankle plantarflexion torque was greater compared with the preferred WR. According to an inverted pendulum model for stance leg movement, a longer step length (higher WR) caused a large vertical displacement of the CoM. The large anteroposterior distance from initial heel contact position to CoM leads to CoM deceleration owing to the effect of the braking GRF [38]. Then, to maintain the walking speed, the propulsive GRF to accelerate the CoM during the late stance phase must increase to recover from the deceleration [11]. The relative shortening of the stance phase time per gait cycle (i.e. reduced duty factor) requires a large vertical GRF to support the body weight. The higher the WR, the greater the GRF components, as mentioned above, suggesting that the increase in the negative power of the hip, knee and ankle during the early stance phase was related to production of the braking GRF and that the increase in the positive power of the ankle during the late stance phase was related to production of the propulsive and vertical GRFs.

The ${\overset{-}{P}}_{\text{total, stance}}$ did not change with a decreased WR (figure 2). Walking at a low WR required less exertion in terms of JT to generate GRF, but the JAV had to be increased, and consequently there was no difference in the ${\overset{-}{P}}_{\text{total, stance}}$. Cavagna & Franzetti [12] reported that the external power to move the CoM decreased at a low WR. However, in our study, there was no difference between the low and preferred WRs when analysed on the basis of the JP.

The hip (sagittal) positive power and the knee negative power increased with increasing cadence in the swing phase. Looking at the pattern of changes in the JP, there were twin peaks in both positive power for the hip (sagittal) and negative power for the knee in the pre-swing and late swing phases, respectively. In addition, the timing of the hip and knee peaks was well matched (figure 3K–N). It is an inefficient form of joint activity for one joint to be performing positive work and the other to be performing negative work at the same time [25]. The positive power of hip flexion torque during the pre- to early swing phase accelerates the thigh forward, and this acceleration acts on the knee joint, causing it to bend. The negative power of knee extension torque during the pre- to early swing phase is required to control this passive knee flexion caused by the hip flexion torque. The positive power of hip extension torque during the late swing phase accelerates the thigh backward, and this acceleration extends the knee joint. The negative power of the knee flexion torque during the late swing phase prevents hyperextension of the knee. These results suggest that the increase in the ${\overset{-}{P}}_{\text{total, swing}}$ at a low WR was caused by the hip and knee JPs being applied strongly and synchronously to prevent excessive knee flexion and extension during fast cadence walking.

The ${\overset{-}{P}}_{\text{total, swing}}$ was significantly reduced for the high WRs (figure 2). This suggests that, at a high WR, the foot of the swinging leg was carried forward as a passive pendulum with relatively little mechanical power input from the muscles of the swinging leg.

The inverse dynamics approach does not assess individual muscles directly. JT is generated by the combined action of multiple muscles, the force application directions and the moment arm of which differ and change over time [25]. We only assessed the relationship between JP and metabolic energy in response to WR. However, this approach has a number of limitations: first, JP is the product of JT and JAV rather than muscle force and velocity. This can result in an overestimation of the amount of muscle-level work performed if storage and release of elastic energy in tendons are present. Second, the amount of mechanical work can overestimate muscle-level work if simultaneous positive and negative joint work are performed at adjacent joints. In this case, two-joint muscles may function to transfer energy between the two adjacent joints. In addition, we only investigated one fixed speed that each participant freely selected as their preferred walking speed. Therefore, it is unclear whether these results can be generalized to other walking speeds. It should also be noted that all participants in this study were healthy young people. In general, older people have a slower walking speed than healthy young people. The main factor in the reduced walking speed with ageing is a reduction in step length while cadence is relatively maintained [39]. The effectiveness of mechanical work production may differ between older and younger people owing to a difference in walking speed. Thus, it will be necessary to expand the current research to a wide range of walking speeds and ages for further progress.

## 5. Conclusion

Our results demonstrate that the WR affects the effectiveness of mechanical work production during walking. The ${\overset{-}{P}}_{\text{total, stance}}$ for the −20% and −15% cadence conditions (i.e. high WR) was significantly higher than that for the preferred WR, while the ${\overset{-}{P}}_{\text{total, swing}}$ for the +20, +15 and +10% cadence conditions (i.e. low WR) was significantly higher than that for the preferred WR. At both high and low WRs, but particularly at a high WR, the lower limb joint must exert a greater amount of mechanical power during walking. In other words, walking at the preferred WR appears to provide optimal effectiveness of mechanical work production, which may help to minimize metabolic energy expenditure. It is clear that the WR is a major parameter in the energetics of human gait.

## Data Availability

All data supporting the findings of this study are available within the article and its online electronic supplementary material [40].
